# 2,2,2-Tri­fluoro­ethyl 5-methyl-1*H*-pyrazole-3-carboxyl­ate

**DOI:** 10.1107/S2414314626000192

**Published:** 2026-01-13

**Authors:** Adriano Bof de Oliveira, Adailton João Bortoluzzi, Alex Fabiani Claro Flores

**Affiliations:** a Escola de Química e Alimentos, Universidade Federal do Rio Grande, Campus Carreiros, 96203-900 Rio Grande-RS, Brazil; b Departamento de Química, Universidade Federal de Santa Catarina, Campus Universitário, 88035-972 Florianópolis-SC, Brazil; University of Aberdeen, United Kingdom

**Keywords:** crystal structure, hydrogen-bonded chain, pyrazole

## Abstract

The title compound, C_7_H_7_F_3_N_2_O_2_, which exhibits disorder over the terminal tri­fluoro­methyl and methyl entities, is close to planar with the r.m.s. deviation for the non-H/-F atoms being 0.038 Å. In the crystal, the mol­ecules are linked by N—H⋯N inter­actions into a [010] chain with a *C*_1_^1^(3) motif. The Hirshfeld surface fingerprint plot analysis indicates that the major contributions for the crystal packing are from H⋯F/F⋯H (31.2%), H⋯H (15.9%), H⋯O/O⋯H (15.3%) and H⋯N/N⋯H (10.1%) contacts.

## Abstract 2

The title compound, which exhibits disorder over the terminal tri­fluoro­methyl and methyl entities, is close to planar with the r.m.s. deviation for the non-H/-F atoms being 0.038 Å. In the crystal, the mol­ecules are linked by N—H⋯N inter­actions into a [010] chain with a *C*_1_^1^(3) motif.

## Graphical abstract

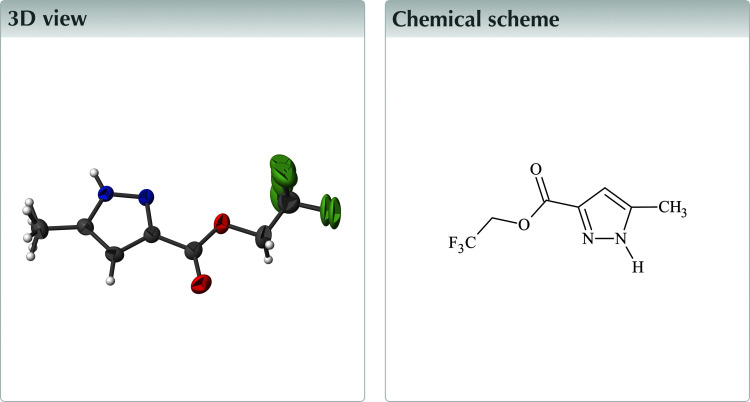

## Structure description

As part of our inter­est in pyrazole derivatives with potential application in medicinal chemistry (Gonçalves *et al.*, 2016 ▸), we now report the crystal structure of the title compound, C_7_H_7_F_3_N_2_O_2_ (**I**). For recent reports regarding pyrazole derivatives, see: Ameziane El Hassani *et al.* (2023 ▸), Ramajayam (2025 ▸) and Ríos & Portilla (2022 ▸).

**Figure 1:**
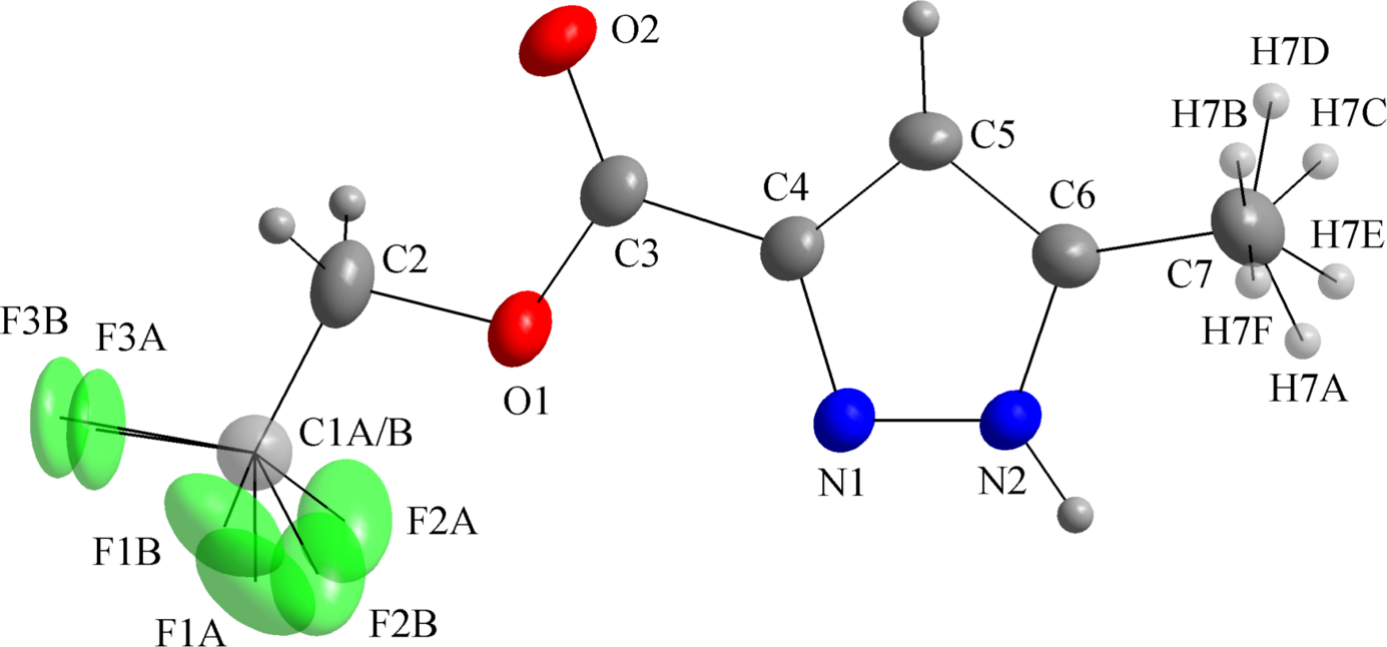
The mol­ecular structure of (**I**) showing displacement ellipsoids drawn at the 40% probability level.

There is one mol­ecule in the asymmetric unit of (**I**), with all atoms being located in general positions (Fig. 1 ▸). The F atoms of the tri­fluoro­methyl entity are disordered over two sets of sites in a 0.718 (11):0.282 (11) ratio and the H atoms of the C7 methyl group are statistically disordered. The mol­ecule is close to planar, with the maximum deviations from the mean plane through the atoms being 0.0746 (17) Å for O1 [r.m.s.d. = 0.038 Å], excluding the hydrogen and the fluorine atoms. The side chain exhibits an extended conformation [C1*A*—C2—O1—C3 = −173.26 (19); C2—O1—C3—C4 = 178.48 (17)°].

**Figure 2:**
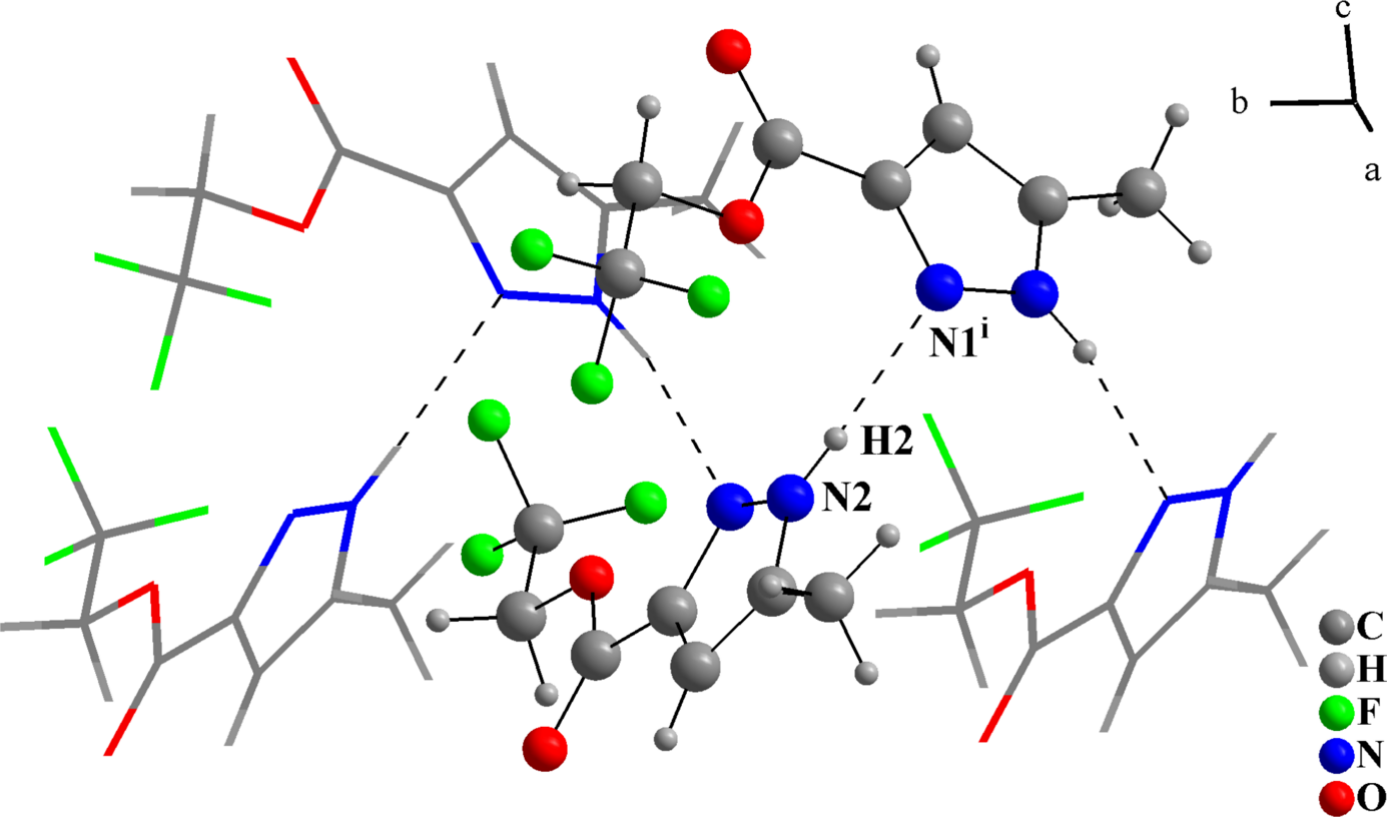
Fragment of the extended structure of (**I**) showing N—H⋯N hydrogen bonds. Symmetry code: (i) −*x* + 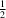, *y* − 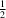, −*z* + 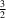; minor disorder components omitted.

**Figure 3:**
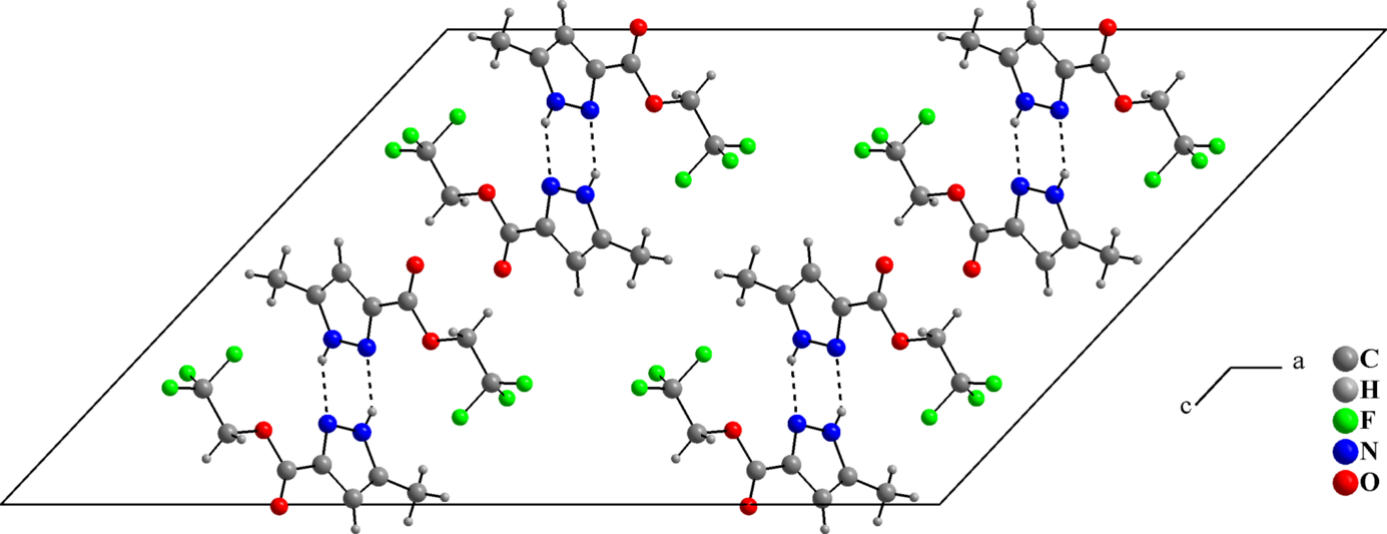
The packing of (**I**) as viewed along the *b*-axis direction.

**Figure 4:**
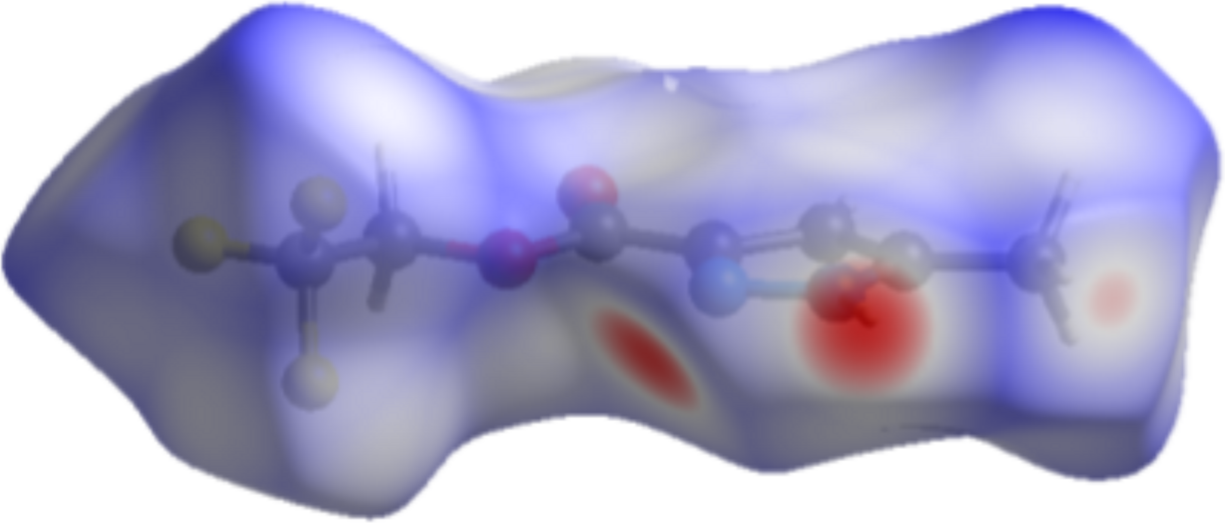
The Hirshfeld surface of (**I**) mapped over *d*_norm_ in the range −0.49 to 1.62 a. u.

**Figure 5:**
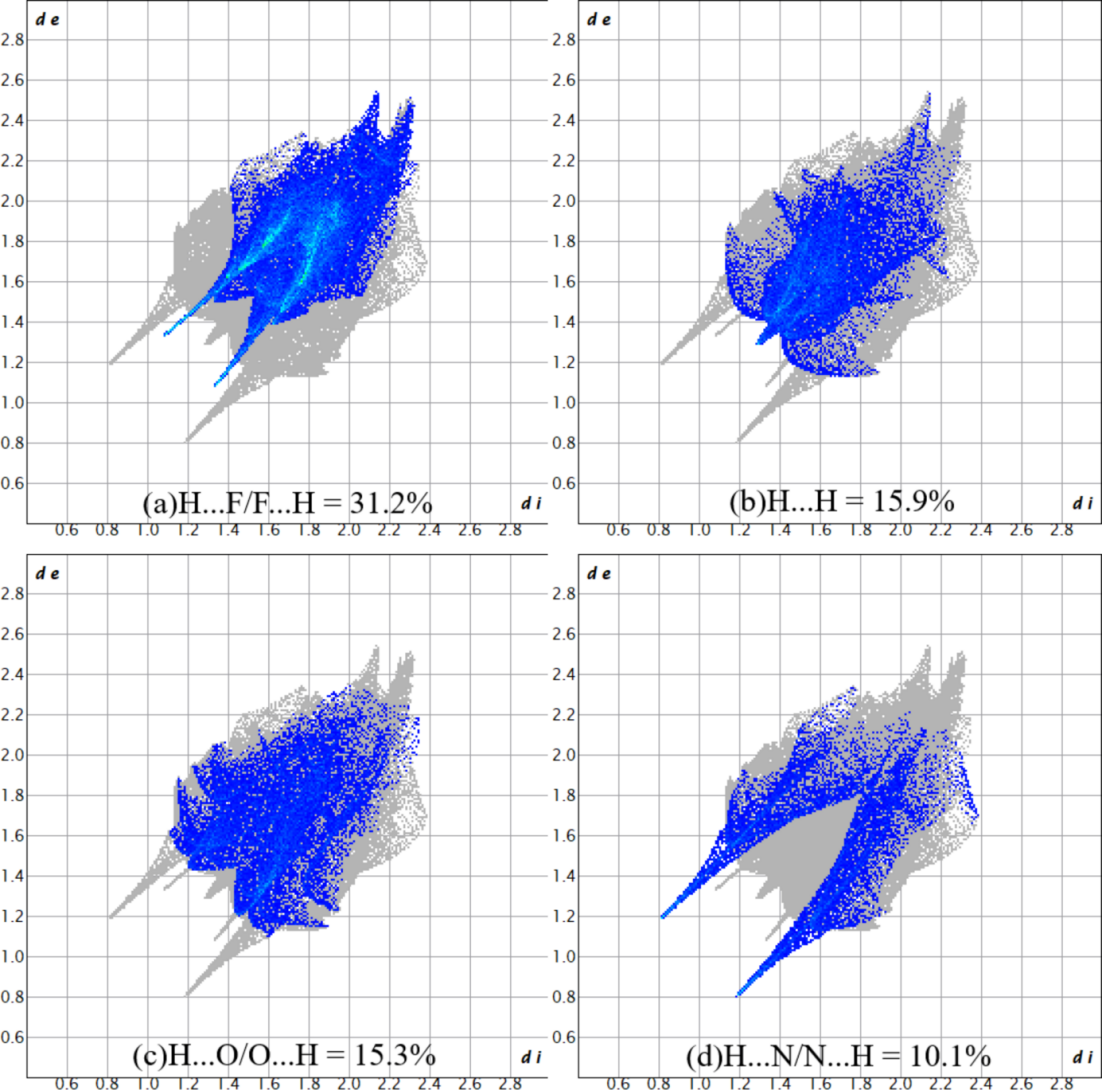
The two-dimensional fingerprint plots for (**I**) for different contact types.

In the crystal, the mol­ecules are connected by N2—H2⋯N1 hydrogen bonds into a ribbon-like chain, which propagates along the *b*-axis direction (Table 1 ▸, Fig. 2 ▸). The crystal structure thus exhibits the supra­molecular arrangement of a catemer with a 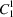
(3) motif (Alkorta *et al.*, 2005 ▸; Foces-Foces *et al.*, 2000 ▸): the ‘up’ and ‘down’ catemers are related by centers of inversion and no strong or relevant inter­actions are observed between the supra­molecular chains (Fig. 3 ▸). There are two short C—F⋯H contacts (Table 1 ▸), but their structure-directing significance is not clear due to the disordered F atoms. The Hirshfeld surface analysis analysis of (**I**) was performed with *Crystal Explorer 21* (Spackman *et al.*, 2021 ▸). The surface mapped over *d*_norm_ shows the regions with the strongest contacts in red in the vicinities of H2 and N1 (Fig. 4 ▸), being in agreement with previous figures (Figs. 2 ▸ and 3 ▸). The fingerprint plots (Fig. 5 ▸) indicate that the H⋯F/F⋯H (31.2%), H⋯H (15.9%), H⋯O/O⋯H (15.3%) and H ⋯N/N⋯H (10.1%) contacts are the most relevant for the crystal cohesion of (**I**).

A survey with the Cambridge Structural Database (CSD, accessed *via* WebCSD on January 2, 2026; Groom *et al.*, 2016 ▸) revealed a similar structure, namely ethyl-5-methyl-1*H*-pyrazole-3-carboxyl­ate, C_7_H_10_N_2_O_2_ (CSD refcode: FAQSAR02; Kusakiewicz-Dawid *et al.*, 2019 ▸). The mol­ecules of FAQSAR02 are linked by C—H⋯O and bifurcated N—H⋯(N,O) inter­actions into a two-dimensional tape-like supra­molecular arrangement. Pyrazole derivatives show distinctive conformations in the solid state: it was pointed out by these authors that methyl and amino pyrazoles lead to structures with the amide or ester substituents and the N—H bond on the opposite side of the five-membered ring (compare C4 and N2 in this work, Fig. 1 ▸). This arrangement was observed in other structures, *e.g.* the derivatives with refcodes: HOKNON and HOKNUT. In contrast, nitro pyrazoles lead to the conformer with the N—H bond on the same side as the amide or carboxyl­ate substituents, as observed in the structures with refcodes: HOKNED and HOKPIJ (Kusakiewicz-Dawid *et al.*, 2019 ▸).

## Synthesis and crystallization

The synthesis and spectroscopic characterization of (**I**) are already published in the literature (Gonçalves *et al.*, 2016 ▸). For the single-crystal X-ray diffractometry reported here, colorless blocks of (**I**) were obtained from a di­chloro­methane solution by slow evaporation of the solvent at room temperature.

## Refinement

Crystal data, data collection and structure refinement details are summarized in Table 2 ▸. The F atoms of the –CF_3_ group are disordered over two sets of sites in a 0.718 (11) (*A* suffix) to 0.282 (11) (*B* suffix) ratio. The H atoms of the C7 methyl group are statistically disordered over two sets of sites.

## Supplementary Material

Crystal structure: contains datablock(s) I. DOI: 10.1107/S2414314626000192/hb4551sup1.cif

Structure factors: contains datablock(s) I. DOI: 10.1107/S2414314626000192/hb4551Isup2.hkl

Supporting information file. DOI: 10.1107/S2414314626000192/hb4551Isup3.cml

CCDC reference: 2521414

Additional supporting information:  crystallographic information; 3D view; checkCIF report

## Figures and Tables

**Table 1:** Hydrogen-bond geometry (Å, °)

*D*—H⋯*A*	*D*—H	H⋯*A*	*D*⋯*A*	*D*—H⋯*A*
N2—H2⋯N1^i^	0.86	2.13	2.922 (2)	153
C7—H7*A*⋯F2*A*^ii^	0.96	2.54	3.477 (7)	165
C7—H7*D*⋯F2*B*^iii^	0.96	2.45	3.394 (11)	168

Symmetry codes: (i) 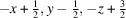; (ii) 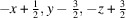; (iii) 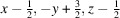.

**Table 2:** Experimental details

Crystal data	
Chemical formula	C_7_H_7_F_3_N_2_O_2_
*M*_r_	208.15
Crystal system, space group	Monoclinic, *C*2/*c*
Temperature (K)	200
*a*, *b*, *c* (Å)	26.914 (6), 5.0133 (10), 18.693 (4)
β (°)	133.214 (2)
*V* (Å^3^)	1838.2 (7)
*Z*	8
Radiation type	Mo *K*α
μ (mm^−1^)	0.15
Crystal size (mm)	0.40 × 0.26 × 0.12

Data collection	
Diffractometer	Bruker APEXII CCD
Absorption correction	Multi-scan (*SADABS*; Krause *et al.*, 2015 ▸)
*T*_min_, *T*_max_	0.603, 0.746
No. of measured, independent and observed [*I* > 2σ(*I*)] reflections	10780, 2644, 1730
*R*_int_	0.033
(sin θ/λ)_max_ (Å^−1^)	0.702

Refinement	
*R*[*F*^2^ > 2σ(*F*^2^)], *wR*(*F*^2^), *S*	0.057, 0.155, 1.04
No. of reflections	2644
No. of parameters	133
H-atom treatment	H-atom parameters constrained
Δρ_max_, Δρ_min_ (e Å^−3^)	0.42, −0.60

Computer programs: *APEX2* and *SAINT* (Bruker, 2014 ▸), *SHELXT2014/5* (Sheldrick, 2015*a* ▸), *SHELXL2019/2* (Sheldrick, 2015*b* ▸), *DIAMOND* (Brandenburg, 2006 ▸), *Crystal Explorer 21* (Spackman *et al.*, 2021 ▸) and *publCIF* (Westrip, 2010 ▸).

## full crystallographic data

### 2,2,2-Trifluoroethyl 5-methyl-1H-pyrazole-3-carboxylate Crystal data

**Table:**

C_7_H_7_F_3_N_2_O_2_	*F*(000) = 848
*M**_r_* = 208.15	*D*_x_ = 1.504 Mg m^−^^3^
Monoclinic, *C*2/*c*	Mo *K*α radiation, λ = 0.71073 Å
*a* = 26.914 (6) Å	Cell parameters from 3527 reflections
*b* = 5.0133 (10) Å	θ = 3.0–29.6°
*c* = 18.693 (4) Å	µ = 0.15 mm^−^^1^
β = 133.214 (2)°	*T* = 200 K
*V* = 1838.2 (7) Å^3^	Prismatic, colourless
*Z* = 8	0.40 × 0.26 × 0.12 mm

### 2,2,2-Trifluoroethyl 5-methyl-1H-pyrazole-3-carboxylate Data collection

**Table:**

Bruker APEXII CCD diffractometer	2644 independent reflections
Radiation source: Fine-focus sealed tube	1730 reflections with *I* > 2σ(*I*)
Horizontally mounted graphite crystal monochromator	*R*_int_ = 0.033
φ and ω scans	θ_max_ = 29.9°, θ_min_ = 2.1°
Absorption correction: multi-scan (SADABS; Krause *et al.*, 2015)	*h* = −37→37
*T*_min_ = 0.603, *T*_max_ = 0.746	*k* = −4→7
10780 measured reflections	*l* = −26→26

### 2,2,2-Trifluoroethyl 5-methyl-1H-pyrazole-3-carboxylate Refinement

**Table:**

Refinement on *F*^2^	Primary atom site location: structure-invariant direct methods
Least-squares matrix: full	Secondary atom site location: difference Fourier map
*R*[*F*^2^ > 2σ(*F*^2^)] = 0.057	Hydrogen site location: inferred from neighbouring sites
*wR*(*F*^2^) = 0.155	H-atom parameters constrained
*S* = 1.04	*w* = 1/[σ^2^(*F*_o_^2^) + (0.050*P*)^2^ + 3.1537*P*] where *P* = (*F*_o_^2^ + 2*F*_c_^2^)/3
2644 reflections	(Δ/σ)_max_ < 0.001
133 parameters	Δρ_max_ = 0.42 e Å^−^^3^
0 restraints	Δρ_min_ = −0.60 e Å^−^^3^

### 2,2,2-Trifluoroethyl 5-methyl-1H-pyrazole-3-carboxylate Special details

**Table:**

Geometry. All esds (except the esd in the dihedral angle between two l.s. planes) are estimated using the full covariance matrix. The cell esds are taken into account individually in the estimation of esds in distances, angles and torsion angles; correlations between esds in cell parameters are only used when they are defined by crystal symmetry. An approximate (isotropic) treatment of cell esds is used for estimating esds involving l.s. planes.

### 2,2,2-Trifluoroethyl 5-methyl-1H-pyrazole-3-carboxylate Fractional atomic coordinates and isotropic or equivalent isotropic displacement parameters (Å^2^)

**Table:**

	*x*	*y*	*z*	*U*_iso_*/*U*_eq_	Occ. (<1)
C2	0.32947 (11)	1.0880 (5)	0.64954 (16)	0.0445 (5)	
H2A	0.309121	1.261903	0.637302	0.053*	
H2B	0.326834	1.046331	0.596343	0.053*	
C3	0.22953 (10)	0.8424 (4)	0.57173 (13)	0.0334 (4)	
C4	0.19776 (9)	0.6313 (4)	0.58381 (13)	0.0298 (4)	
C5	0.13094 (9)	0.5346 (4)	0.51236 (13)	0.0351 (5)	
H5	0.097239	0.590858	0.447569	0.042*	
C6	0.12582 (9)	0.3380 (4)	0.55871 (13)	0.0335 (4)	
C7	0.06852 (11)	0.1606 (5)	0.52476 (17)	0.0483 (6)	
H7A	0.085442	0.026166	0.573408	0.072*	0.5
H7B	0.048965	0.076990	0.464084	0.072*	0.5
H7C	0.034334	0.264684	0.514954	0.072*	0.5
H7D	0.027052	0.219061	0.461556	0.072*	0.5
H7E	0.063529	0.168237	0.570880	0.072*	0.5
H7F	0.078160	−0.019457	0.520010	0.072*	0.5
C1A	0.40082 (14)	1.0874 (6)	0.7439 (2)	0.0594 (7)*	0.718 (11)
F1A	0.4328 (3)	0.8643 (15)	0.7764 (6)	0.113 (2)	0.718 (11)
F2A	0.4018 (3)	1.1767 (14)	0.8164 (3)	0.0867 (16)	0.718 (11)
F3A	0.4367 (5)	1.253 (3)	0.7457 (9)	0.0838 (11)	0.718 (11)
C1B	0.40082 (14)	1.0874 (6)	0.7439 (2)	0.0594 (7)*	0.282 (11)
F1B	0.4233 (9)	0.839 (5)	0.7329 (14)	0.113 (2)	0.282 (11)
F2B	0.4209 (7)	1.060 (3)	0.8212 (8)	0.0867 (16)	0.282 (11)
F3B	0.4429 (15)	1.288 (8)	0.741 (2)	0.0838 (11)	0.282 (11)
N1	0.23275 (8)	0.5042 (4)	0.66983 (10)	0.0303 (4)	
N2	0.18769 (7)	0.3269 (3)	0.65185 (10)	0.0307 (4)	
H2	0.197260	0.217450	0.695247	0.037*	
O1	0.29450 (7)	0.8889 (3)	0.65623 (10)	0.0399 (4)	
O2	0.20159 (8)	0.9569 (3)	0.49600 (11)	0.0478 (4)	

### 2,2,2-Trifluoroethyl 5-methyl-1H-pyrazole-3-carboxylate Atomic displacement parameters (Å^2^)

**Table:**

	*U*^11^	*U*^22^	*U*^33^	*U*^12^	*U*^13^	*U*^23^
C2	0.0536 (12)	0.0447 (14)	0.0480 (12)	−0.0159 (11)	0.0397 (11)	−0.0078 (10)
C3	0.0428 (10)	0.0333 (12)	0.0332 (9)	−0.0012 (9)	0.0295 (8)	−0.0024 (8)
C4	0.0354 (9)	0.0313 (11)	0.0277 (8)	−0.0008 (8)	0.0236 (7)	−0.0017 (7)
C5	0.0330 (9)	0.0402 (13)	0.0251 (8)	0.0027 (9)	0.0172 (7)	0.0028 (8)
C6	0.0306 (9)	0.0360 (12)	0.0298 (8)	−0.0005 (8)	0.0191 (8)	−0.0012 (8)
C7	0.0359 (10)	0.0500 (15)	0.0482 (12)	−0.0071 (10)	0.0246 (10)	−0.0014 (11)
F1A	0.054 (2)	0.089 (2)	0.130 (5)	0.0057 (18)	0.038 (3)	0.018 (4)
F2A	0.088 (3)	0.121 (4)	0.0569 (11)	−0.051 (3)	0.0515 (16)	−0.045 (2)
F3A	0.070 (2)	0.095 (4)	0.0957 (18)	−0.0487 (18)	0.0607 (14)	−0.012 (2)
F1B	0.054 (2)	0.089 (2)	0.130 (5)	0.0057 (18)	0.038 (3)	0.018 (4)
F2B	0.088 (3)	0.121 (4)	0.0569 (11)	−0.051 (3)	0.0515 (16)	−0.045 (2)
F3B	0.070 (2)	0.095 (4)	0.0957 (18)	−0.0487 (18)	0.0607 (14)	−0.012 (2)
N1	0.0332 (7)	0.0330 (9)	0.0267 (7)	−0.0038 (7)	0.0213 (6)	−0.0016 (6)
N2	0.0326 (7)	0.0330 (10)	0.0263 (7)	−0.0019 (7)	0.0200 (6)	0.0028 (6)
O1	0.0445 (8)	0.0450 (10)	0.0343 (7)	−0.0127 (7)	0.0286 (6)	−0.0036 (6)
O2	0.0564 (9)	0.0490 (11)	0.0391 (8)	−0.0020 (8)	0.0331 (7)	0.0102 (7)

### 2,2,2-Trifluoroethyl 5-methyl-1H-pyrazole-3-carboxylate Geometric parameters (Å, º)

**Table:**

C2—O1	1.435 (2)	C7—H7A	0.9600
C2—C1A	1.470 (4)	C7—H7B	0.9600
C2—C1B	1.470 (4)	C7—H7C	0.9600
C2—H2A	0.9700	C7—H7D	0.9600
C2—H2B	0.9700	C7—H7E	0.9600
C3—O2	1.201 (2)	C7—H7F	0.9600
C3—O1	1.351 (2)	C1A—F3A	1.256 (7)
C3—C4	1.472 (3)	C1A—F1A	1.283 (8)
C4—N1	1.343 (2)	C1A—F2A	1.412 (6)
C4—C5	1.401 (3)	C1B—F2B	1.155 (11)
C5—C6	1.380 (3)	C1B—F1B	1.46 (3)
C5—H5	0.9300	C1B—F3B	1.539 (18)
C6—N2	1.355 (2)	N1—N2	1.347 (2)
C6—C7	1.494 (3)	N2—H2	0.8600
			
O1—C2—C1A	106.9 (2)	C6—C7—H7E	109.5
O1—C2—C1B	106.9 (2)	H7A—C7—H7E	56.3
O1—C2—H2A	110.3	H7B—C7—H7E	141.1
C1A—C2—H2A	110.3	H7C—C7—H7E	56.3
O1—C2—H2B	110.3	H7D—C7—H7E	109.5
C1A—C2—H2B	110.3	C6—C7—H7F	109.5
H2A—C2—H2B	108.6	H7A—C7—H7F	56.3
O2—C3—O1	124.09 (19)	H7B—C7—H7F	56.3
O2—C3—C4	124.35 (18)	H7C—C7—H7F	141.1
O1—C3—C4	111.56 (16)	H7D—C7—H7F	109.5
N1—C4—C5	111.54 (17)	H7E—C7—H7F	109.5
N1—C4—C3	121.32 (16)	F3A—C1A—F1A	108.1 (8)
C5—C4—C3	127.14 (17)	F3A—C1A—F2A	104.8 (7)
C6—C5—C4	105.27 (16)	F1A—C1A—F2A	104.8 (4)
C6—C5—H5	127.4	F3A—C1A—C2	112.3 (6)
C4—C5—H5	127.4	F1A—C1A—C2	118.2 (4)
N2—C6—C5	105.83 (17)	F2A—C1A—C2	107.5 (3)
N2—C6—C7	121.63 (19)	F2B—C1B—F1B	101.1 (9)
C5—C6—C7	132.54 (18)	F2B—C1B—C2	127.5 (7)
C6—C7—H7A	109.5	F1B—C1B—C2	99.2 (7)
C6—C7—H7B	109.5	F2B—C1B—F3B	113.0 (14)
H7A—C7—H7B	109.5	F1B—C1B—F3B	99.9 (19)
C6—C7—H7C	109.5	C2—C1B—F3B	110.3 (13)
H7A—C7—H7C	109.5	C4—N1—N2	103.93 (15)
H7B—C7—H7C	109.5	N1—N2—C6	113.44 (16)
C6—C7—H7D	109.5	N1—N2—H2	123.3
H7A—C7—H7D	141.1	C6—N2—H2	123.3
H7B—C7—H7D	56.3	C3—O1—C2	114.79 (16)
H7C—C7—H7D	56.3		
			
O2—C3—C4—N1	177.56 (19)	O1—C2—C1B—F1B	73.5 (9)
O1—C3—C4—N1	−1.6 (3)	O1—C2—C1B—F3B	177.7 (17)
O2—C3—C4—C5	−1.9 (3)	C5—C4—N1—N2	0.3 (2)
O1—C3—C4—C5	178.99 (18)	C3—C4—N1—N2	−179.26 (16)
N1—C4—C5—C6	0.0 (2)	C4—N1—N2—C6	−0.5 (2)
C3—C4—C5—C6	179.51 (19)	C5—C6—N2—N1	0.5 (2)
C4—C5—C6—N2	−0.3 (2)	C7—C6—N2—N1	−178.95 (18)
C4—C5—C6—C7	179.1 (2)	O2—C3—O1—C2	−0.6 (3)
O1—C2—C1A—F3A	179.1 (8)	C4—C3—O1—C2	178.48 (17)
O1—C2—C1A—F1A	52.1 (5)	C1A—C2—O1—C3	−173.26 (19)
O1—C2—C1A—F2A	−66.1 (4)	C1B—C2—O1—C3	−173.26 (19)
O1—C2—C1B—F2B	−38.3 (12)		

### 2,2,2-Trifluoroethyl 5-methyl-1H-pyrazole-3-carboxylate Hydrogen-bond geometry (Å, º)

**Table:**

*D*—H···*A*	*D*—H	H···*A*	*D*···*A*	*D*—H···*A*
N2—H2···N1^i^	0.86	2.13	2.922 (2)	153
C7—H7*A*···F2*A*^ii^	0.96	2.54	3.477 (7)	165
C7—H7*D*···F2*B*^iii^	0.96	2.45	3.394 (11)	168

Symmetry codes: (i) −*x*+1/2, *y*−1/2, −*z*+3/2; (ii) −*x*+1/2, *y*−3/2, −*z*+3/2; (iii) *x*−1/2, −*y*+3/2, *z*−1/2.

### Hydrogen-bond geometry (Å, °) and selected torsion angles (°) for the ethyl 5-methyl-1*H*-pyrazole-3-carboxylate derivative (Figs. 11 and 12; refcode: FAQSAR02; Kusakiewicz-Dawid *et al.*, 2019)

**Table:**

*D*—H···*A*	*D*—H	H···*A*	*D*···*A*	*D*—H···*A*
C4—H4*C*···O1^i^	0.96	2.66	3.5453 (19)	153.9
N1—H1···N2^ii^	0.86	2.17	2.9852 (18)	157.2
N1—H1···O2^ii^	0.86	2.48	3.0998 (16)	129.3
				
Atom chains	Torsion angles		Atom chains	Torsion angles
C1/C2/C3/C4	179.66 (16)		C2/C1/C5/O1	-5.5 (3)
C1/C5/O2/C6	-179.37 (12)		C2/C1/C5/O2	173.72 (14)
C1/N2/N1/C3	-0.54 (16)		C5/O2/C6/C7	174.94 (12)

Symmetry codes: (i) -*x*+1/2, *y*+1/2, -*z*+1/2; (ii) -*x*+3/2, *y*+1/2, -*z*+1/2.
